# Supplementary material for: The glutamate/cystine xCT antiporter antagonizes glutamine metabolism and reduces nutrient flexibility
Source: Nat Commun. 2017 Apr 21;8:15074. doi: 10.1038/ncomms15074 (PMC5413954; doi:10.1038/ncomms15074)
Supplement: Supplementary Information — Supplementary Figures [file ncomms15074-s1.pdf]

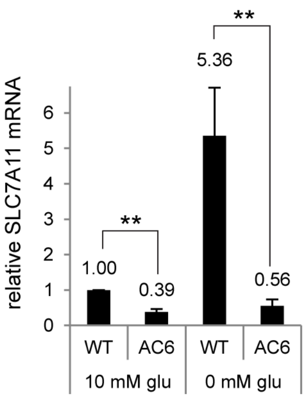

Supplementary Figure 1. RNA analysis of *SLC7A11* gene-trap clone

SLC7A11 mRNA levels measured by real-time PCR in wildtype Hap1 (WT) and AC6 (SLC7A11 gene-trap clone) cells. Cells were cultured with in media with the indicated glucose concentration for 16 hr. GAPDH was used for real-time RT-PCR normalization. Data represent the means ± *s.d.* ($n=3$); **, $p<0.01$; unpaired Student's t-test.

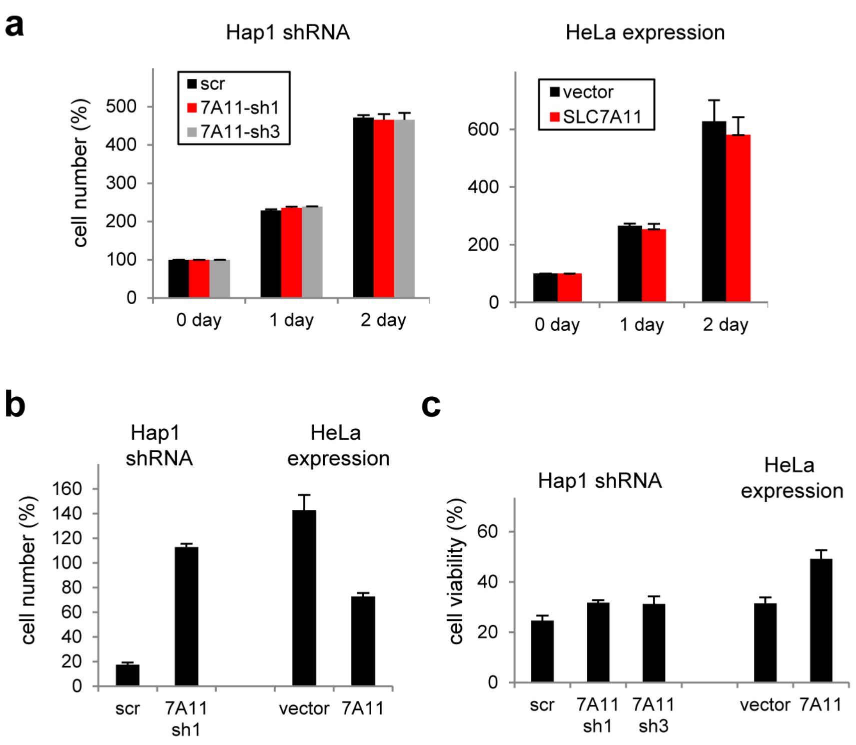

Supplementary Figure 2. Proliferation and cell viability assays for *SLC7A11*-knockdown Hap1 and *SLC7A11*-overexpressing HeLa cells

(**a**) Proliferation rates in glucose-replete media. The medium was DMEM A14430 containing 10% dialyzed FBS, 10 mM glucose and 6 mM glutamine. Data represent the means ± *s.d.* (*n=3*). (**b**) Cell viability in low glucose media. Cell were cultured in DMEM A14430 containing 10% dialyzed FBS, 0.2 mM glucose and 1 mM glutamine for 2 days. Data represent the means ± *s.d.* (*n=3*). (**c**) Viability in glutamine-deficient media. Cells were cultured in glutamine-deficient medium (DMEM A14430 containing 10% dialyzed FBS, 10 mM glucose and 0 mM glutamine) for 3 days and cell viability was determined. Data represent the means ± *s.d.* (*n=3*).

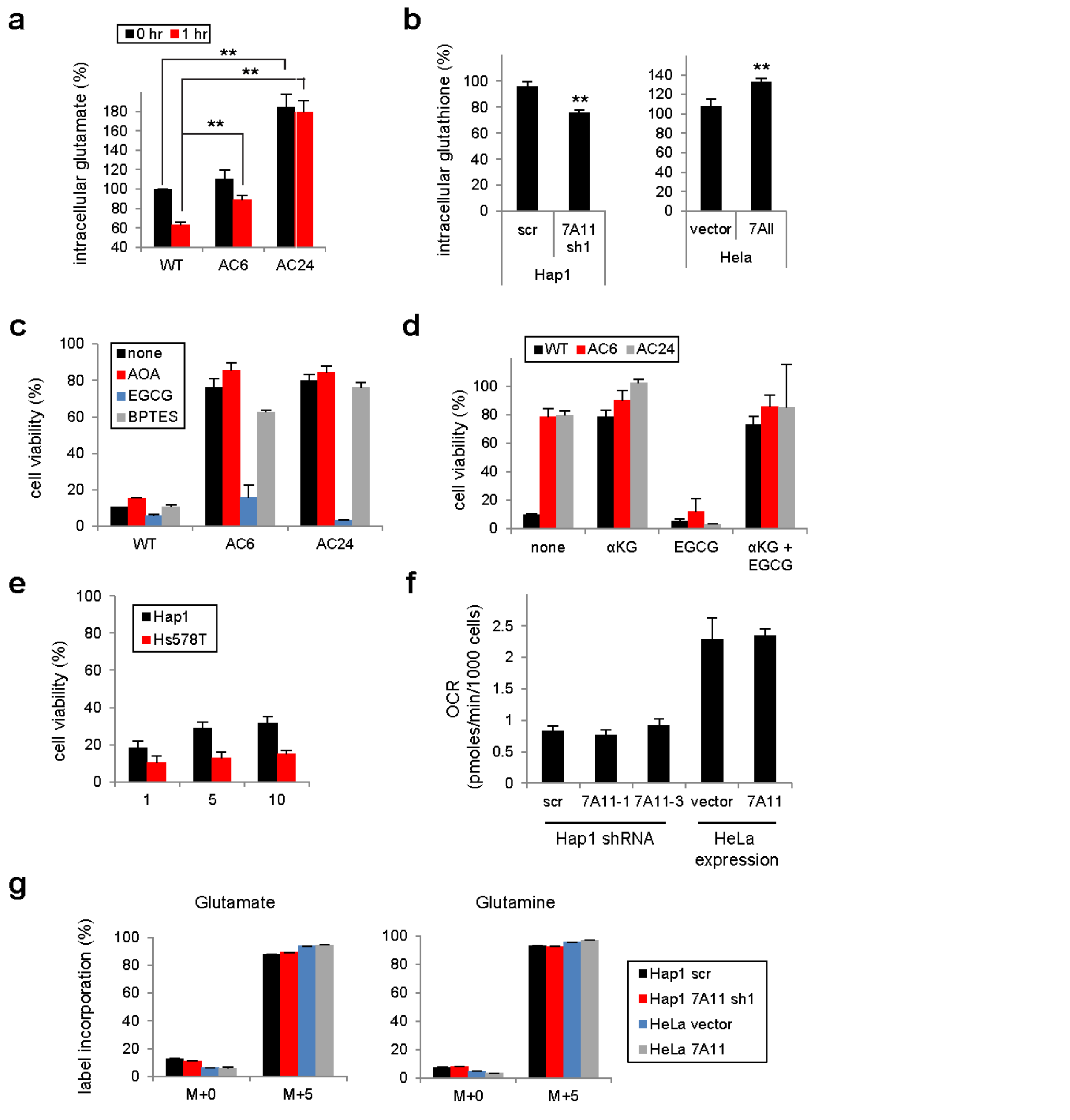

Supplementary Figure 3. Glutamate, glutathione, cell viability and respiration assays

(**a**) Quantification of intracellular glutamate of Hap1 gene-trap mutants before and one hour after glucose removal. Data represents the mean ± *s.d.* (*n*=3); **, *p*<0.01; unpaired Student's t-test. (**b**) Quantification of intracellular total glutathione of Hap1 and HeLa cells at 1 hour after glucose removal. Data represents the mean ± *s.d.* (*n*=3); **, *p*<0.01; unpaired Student's t-test. (**c**, **d**) Cell

viability at 24 hr after glucose withdrawal. The following drugs were added as indicated: 0.5 mM AOA, 50 μM EGCG, 10 μM BPTES or 4 mM dm-αKG. Data represents the mean ± *s.d.* (*n=3*). (**e**) Viability of Hap1 and Hs578T cells in glucose-deficient medium with varying glutamine supplementation. Cells were incubated with medium (DMEM A14430 containing 10% dialyzed FBS, 0 mM glucose, and 1, 5 or 10 mM glutamine) for 24 hr and cell viability was determined. Data represent the means ± *s.d.* (*n=3*). (**f**) Oxygen consumption under glucose-replete conditions. OCR in SLC7A11 knockdown Hap1 cells and SLC7A11-overexpressing HeLa cells was measured in the presence of 10 mM glucose + 6 mM glutamine. Data represents the mean ± *s.d.* (*n=4*). (**g**) Metabolic labeling with isotope-labeled glutamine. Cells were incubated for 8 hr in glucose free media containing U-$^{13}C_5$-glutamine before extracting metabolites. Data represent the means ± *s.d.* (*n=3*).

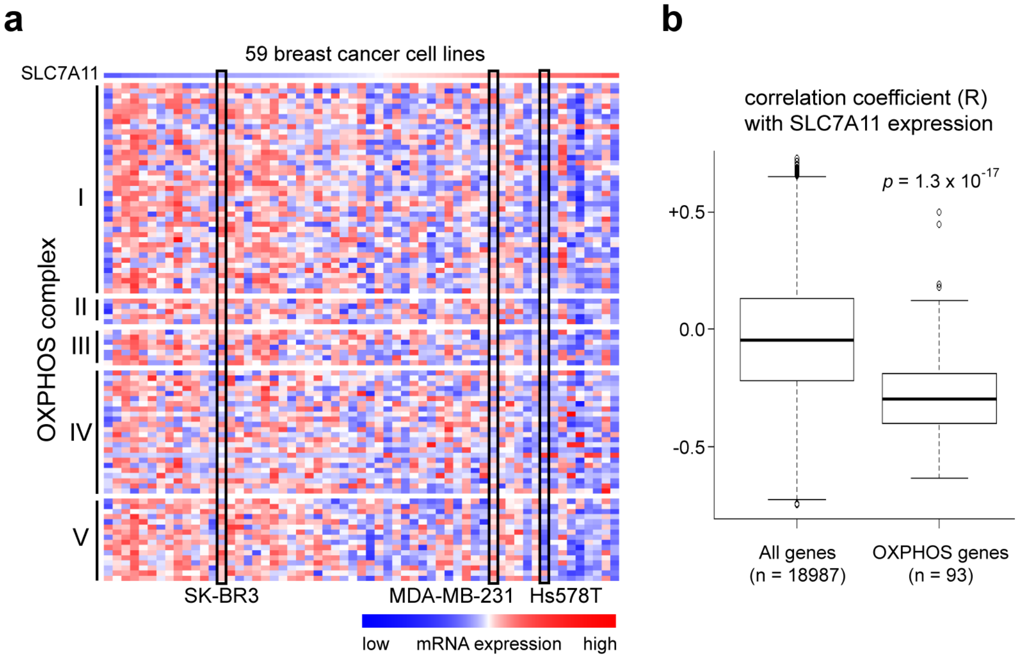

Supplementary Figure 4. Correlation of *SLC7A11* and OXPHOS gene expression in breast cancer cell lines

(**a**) Heatmap of gene expression values of *SLC7A11* and OXPHOS complex genes across 59 breast cancer cells in the CCLE expression data. 59 breast cancer cells are arranged in the order of SLC7A11 expression level from lowest (blue) to highest (red). (**b**) Correlation coefficient (R) between expression of *SLC7A11* and that of all other genes (n = 18987) or OXPHOS genes (n = 93) across 59 breast cancer cells. The two-sided Student's *t* test was used to calculate *p* values.

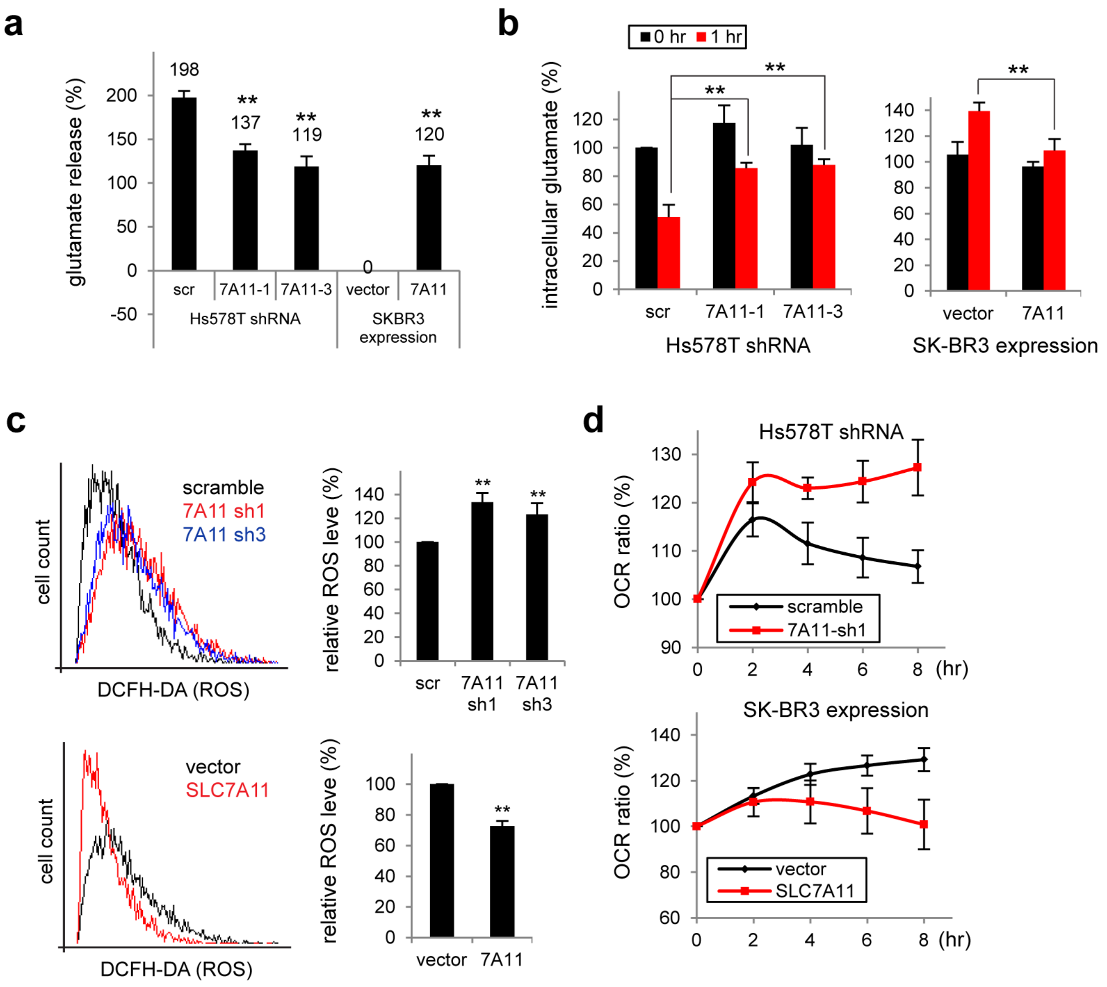

Supplementary Figure 5. SLC7A11 expression and ROS level in breast cancer cells

(**a**) Glutamate release into the medium. All values were normalized to glutamate release of Hap1 control cells shown in Fig. 2b. Data represent the means ± *s.d.* (*n=3*); **, *p*<0.01; unpaired Student's t-test. (**b**) Intracellular glutamate levels before and 1 hr after glucose removal. Values are normalized to control cells at 0 hr. Data represent the means ± *s.d.* (*n=3*); **, *p*<0.01; unpaired Student's t-test. (**c**) Cellular ROS levels in *SLC7A11* knockdown and *SLC7A11*-overexpressing Hs578T cells determined by flow cytometer analysis of 10 μM 2', 7'-dichlorofluorescin diacetate (DCFH-DA) fluorescence. Cells were cultured in DMEM A14430 containing 10% dialyzed FBS, 10 mM glucose and 2 mM glutamine. The right panel data

represent the means ± *s.d*. (*n=3*); **, $p<0.01$; unpaired Student's t-test. (**d**) Maintenance of respiratory chain activity upon glucose withdrawal. Oxygen consumption was determined at 2 hr intervals after glucose withdrawal, in the presence of 1 mM glutamine. Data represent the means ± *s.d*. (*n=3*).

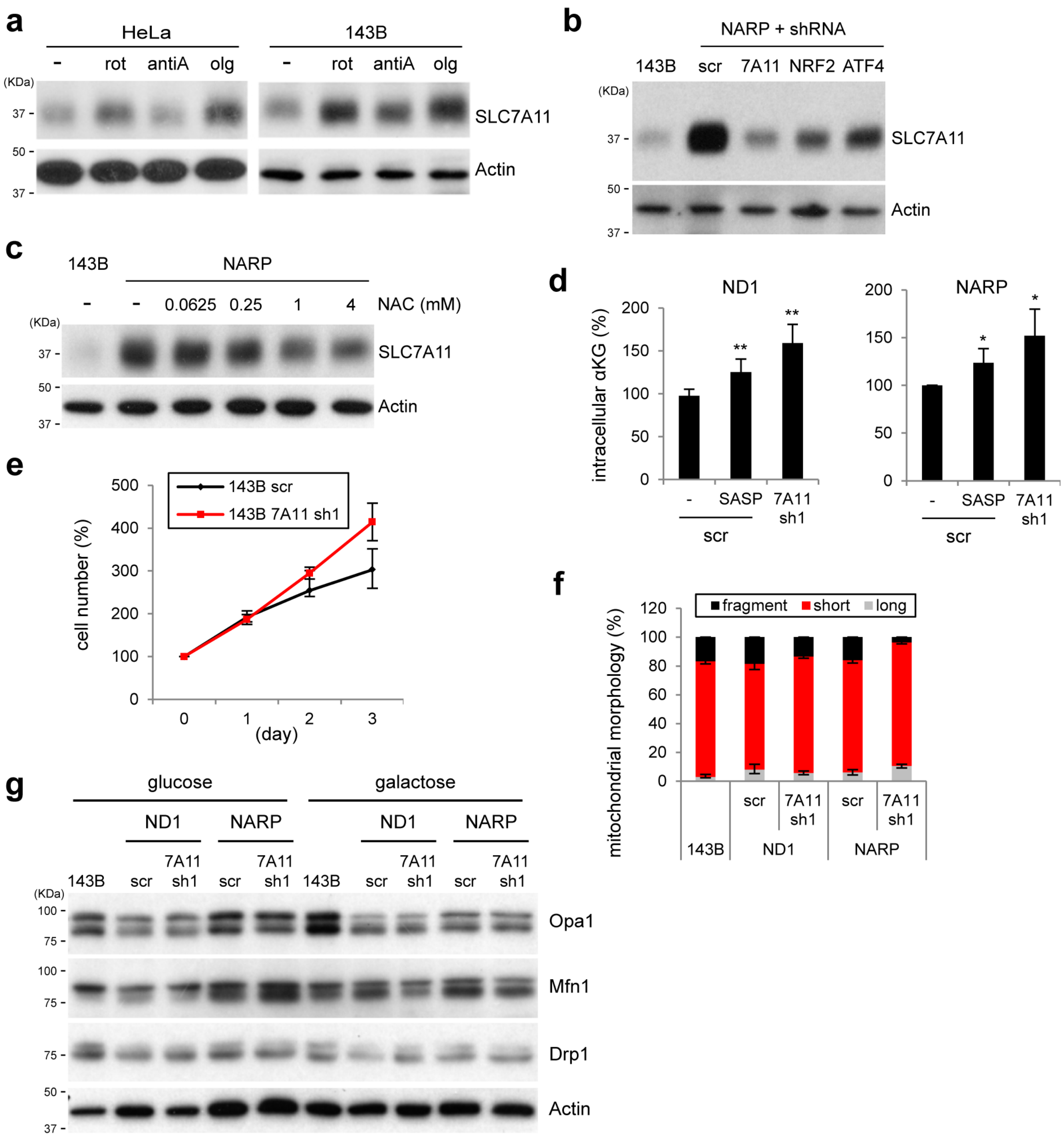

Supplementary Figure 6. Analysis of cybrids

(**a**) Western blot of SLC7A11 levels in HeLa and 143B cells treated with rotenone (1 μM), oligomycin (5 μM) or antimycin A (5 μM). (**b**, **c**) Western blot of SLC7A11 levels in *SLC7A11*, *Nrf2* and *Atf4*-knockdown or N-acetylcysteine-treated NARP cybrid cells. (**d**) Quantification of intracellular α-ketoglutarate at 6 hr after galactose culture. *SLC7A11* knockdown or

sulfasalazine (SASP)-treated (500 μM) ND1 and NARP cells were cultured in the presence of 10 mM galactose + 2 mM glutamine. Data represents the mean ± *s.d.* ($n=3$); *, $p<0.05$. **, $p<0.01$; unpaired Student's t-test. (**e**) Proliferation rates of control and *SLC7A11* knockdown 143B cells. Cells were cultured in galactose medium (DMEM A14430 containing 10% dialyzed FBS, 10 mM galactose and 2 mM glutamine) for 3 days. Data represent the means ± *s.d.* ($n=4$). (**f**) Quantification of mitochondrial morphology. Cells were cultured in the presence of 10 mM glucose + 2 mM glutamine. Data represent the means ± *s.d.* ($n=3$). (**g**) Western blot of mitochondrial dynamics proteins. Cells were cultured in the presence of 10 mM glucose or galactose + 2 mM glutamine for 24 hr.

Fig 1b

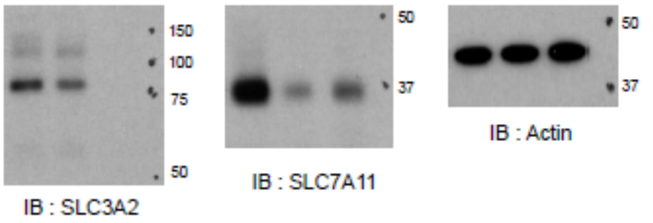

Fig 2a & 4a

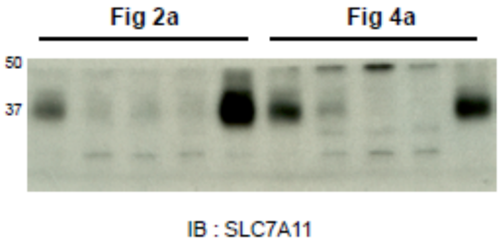

Fig 5e

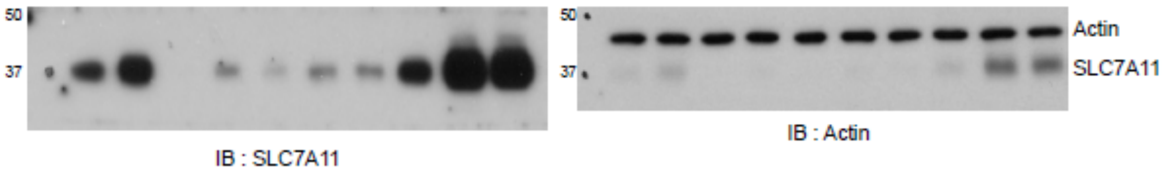

Fig 6a

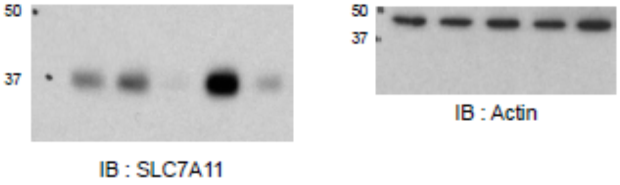

Supplementary Figure 7. Uncropped scans of key Western blots
